# Non-small cell lung cancer: Whole-lesion histogram analysis of the apparent diffusion coefficient for assessment of tumor grade, lymphovascular invasion and pleural invasion

**DOI:** 10.1371/journal.pone.0172433

**Published:** 2017-02-16

**Authors:** Naoko Tsuchiya, Mariko Doai, Katsuo Usuda, Hidetaka Uramoto, Hisao Tonami

**Affiliations:** 1 Department of Radiology, Kanazawa Medical University, Uchinada, Ishikawa, Japan; 2 Department of Thoracic Surgery, Kanazawa Medical University, Uchinada, Ishikawa, Japan; Seconda Universita degli Studi di Napoli, ITALY

## Abstract

**Purpose:**

Investigating the diagnostic accuracy of histogram analyses of apparent diffusion coefficient (ADC) values for determining non-small cell lung cancer (NSCLC) tumor grades, lymphovascular invasion, and pleural invasion.

**Materials and methods:**

We studied 60 surgically diagnosed NSCLC patients. Diffusion-weighted imaging (DWI) was performed in the axial plane using a navigator-triggered single-shot, echo-planar imaging sequence with prospective acquisition correction. The ADC maps were generated, and we placed a volume-of-interest on the tumor to construct the whole-lesion histogram. Using the histogram, we calculated the mean, 5th, 10th, 25th, 50th, 75th, 90th, and 95th percentiles of ADC, skewness, and kurtosis. Histogram parameters were correlated with tumor grade, lymphovascular invasion, and pleural invasion. We performed a receiver operating characteristics (ROC) analysis to assess the diagnostic performance of histogram parameters for distinguishing different pathologic features.

**Results:**

The ADC mean, 10th, 25th, 50th, 75th, 90th, and 95th percentiles showed significant differences among the tumor grades. The ADC mean, 25th, 50th, 75th, 90th, and 95th percentiles were significant histogram parameters between high- and low-grade tumors. The ROC analysis between high- and low-grade tumors showed that the 95th percentile ADC achieved the highest area under curve (AUC) at 0.74. Lymphovascular invasion was associated with the ADC mean, 50th, 75th, 90th, and 95th percentiles, skewness, and kurtosis. Kurtosis achieved the highest AUC at 0.809. Pleural invasion was only associated with skewness, with the AUC of 0.648.

**Conclusions:**

ADC histogram analyses on the basis of the entire tumor volume are able to stratify NSCLCs' tumor grade, lymphovascular invasion and pleural invasion.

## Introduction

Lung cancer is the most common malignant tumor and has become the main cause of cancer mortality [1]. From a clinical point of view, it is important to predict tumor aggressiveness in order to select the proper treatment strategy. Positron emission tomography using ^18^F-fluorodeoxyglucose (^18^F-FDG PET) has been used for evaluating the tumor aggressiveness of non-small cell lung cancer (NSCLC) [2,3], but ^18^F-FDG PET is not widely used because of its high cost. The use of lung magnetic resonance (MR) imaging is gradually increasing in clinical practice, not only because of its lower cost, but also due to its easy applicability for various pathologic conditions.

Recent developments in diffusion-weighted imaging (DWI) have addressed its potential advantages and applications for the characterization of lung cancer [4,5]. The diffusion coefficient of water in living tissue calculated by an MR examination is expressed as the apparent diffusion coefficient (ADC). Some studies have suggested that the ADC could be used to demonstrate the histological characteristics of lung cancers, and that it may be useful for distinguishing the degree of cell differentiation [6–11]. However, the averaged mean ADC is calculated from the largest slice of a tumor, and thus the mean ADC may not represent the full spectrum of histology within a tumor.

Theoretically, an ADC histogram can display ADC values and their distribution within a whole tumor, and such a histogram could be used to analyze the ADC voxel by voxel, thereby providing more precise information than the mean ADC [12]. To the best of our knowledge, there has not been a study that assessed the value of ADC histograms for predicting the aggressiveness of lung cancer. On the other hand, DWI of the lung is technically challenging and not always feasible due to shortcomings such as motion artifacts associated with air-tissue interfaces [4]. Respiratory triggering by the navigator-echo method has recently been explored [13]. The use of the navigator-echo enables decrease number of ghosting and artifacts, and improves the quality of DW images [14].

The purpose of the present study was to investigate the diagnostic accuracy of histogram analyses of ADC values acquired by respiratory triggering and the navigator-echo method for determining the tumor grade, lymphovascular invasion and pleural invasion of NSCLC.

## Materials and methods

### Patients

The institutional ethics committee of Kanazawa Medical University approved this retrospective study and waived the requirement for informed consent (approval number 1003). Between January 2012 and December 2014, 166 patients with NSCLC underwent DWI followed by surgical resection. Of these, tumors measuring ≤10 mm in diameter (n = 21), tumors with predominant ground-glass opacity (GGO) (n = 15), tumors with large cystic change or necrosis (n = 9), and tumors with obstructive pneumonia or air- containing cavity (n = 51) based on their computed tomography (CT) appearance were excluded from the analysis (S1 Fig). Patients whose DW images showed poor image quality resulting from motion or a severe magnetic susceptibility artifact (n = 10) were also excluded from the data analysis.

As a result, 60 patients (37 males and 23 females, mean age 75.7 years, age range 56–88 years) were enrolled in this study. Table 1 shows the patient and tumor characteristics, including patient age, tumor size, histologic type, pathological grade, lymphovascular invasion, and pleural invasion.

**Table 1:** NSCLC Patient and Tumor Characteristics.

Characteristics (n = 60)	Value
Age (yr)	
Mean (±SD)	75.7 (7.0)
Range	56–88
Tumor size (mm)	
Mean (±SD)	34.9 (14.4)
Range	12–57
Histologic type	
Squamous cell carcinoma	21
Adenocarcinoma	38
Large cell carcinoma	1
Pathological grade	
Grade 1	15
Grade 2	30
Grade 3	15
Lymphovascular invasion	
Positive	49
Negative	11
Pleural invasion	
Positive	30
Negative	30

### DWI

All MR examinations were performed using a 1.5-T system (Magnetom Avanto, Siemens, Erlangen, Germany) with a 45 mT/m gradient strength, and a 32-channel body phased-array coil. DWI was acquired using a free-breathing navigator-triggered single-shot, echo-planar imaging sequence with prospective acquisition correction (PACE) in the axial plane as described in detail previously [14]. The data were acquired in the end-expiratory phase. The sequence parameters were as follows: TR 4000–6000 ms (equal to the respiratory cycle of the patient), TE 65 ms, acquisition matrix 128 × 96 (interpolated to 256 × 192), field of view 262 × 350 mm, section thickness of 6-mm with a 2-mm intersection gap, 35 slices by four concatenations, four averages, 144 EPI factors, spectral fat-suppression, parallel imaging with the acceleration factor of 2, and tridimensional gradients with b-values of 0 and 800 s/mm^2^. The acquisition time for PACE DWI was approx. 4–5 min depending on the respiratory cycle.

### Image analysis

**Fig 1:**
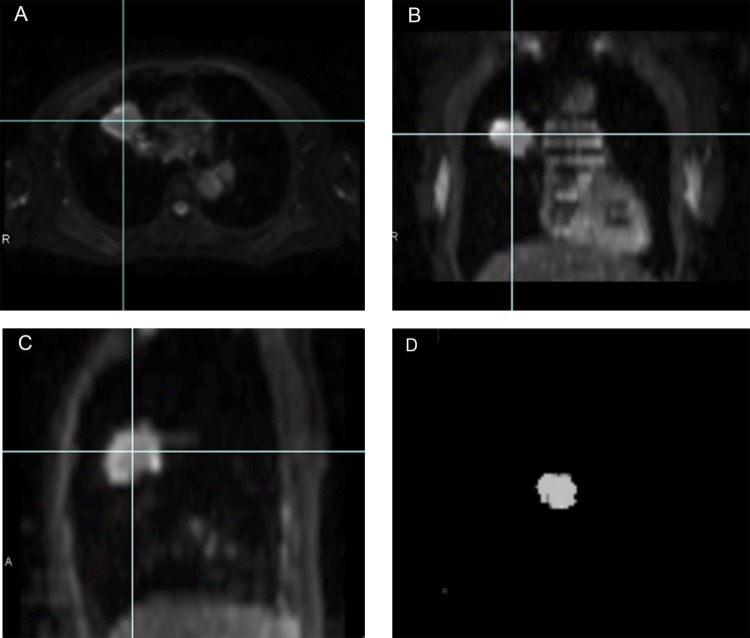
A 67-year-old woman with adenocarcinoma of the right upper lobe. The outline of the tumor is drawn on the axial low-b value DW image (b = 0 s/mm^2^) (A). The tumor's boundaries are meticulously identified with reference to the coronal and sagittal reformatted DW images (B, C). The data acquired from each slice are summed to generate volumes of interest (VOIs) (D). The contours of the VOI are automatically copied to the exact same location of the corresponding ADC maps.

The ADC maps were generated automatically from each DW image by the MR system software. Regions of interest (ROIs) were manually traced just inside the outer edge of the tumor on all slices of low-b value (b = 0 s/mm^2^) DW images using Ziostation 2 (Ziosoft, Tokyo) by two radiologists (N.T. and M.D., with 5 and 10 years’ experience in chest MR imaging). The data acquired from each slice were summed to generate volumes of interest (VOIs). The contours of each VOI were automatically copied to the exact same location of the corresponding ADC maps. In this way, more accurate VOIs can be obtained compared to those drawn directly on ADC maps. An example of the process of extracting the VOI of a tumor is shown in Fig 1.

All ADC values within the VOI were used to compute the average ADC within the tumor. The ADC values were then binned to construct the ADC histogram. The following parameters were calculated from the ADC histogram: (a) mean ADC; (b) 5th, 10th, 25th, 50th, 75th, 90th, and 95th percentiles of the ADC values; (c) skewness; (d); and kurtosis. The *n*th percentile was the point at which *n*% of the voxel values from that histogram were observed on the left. Skewness reflects the shift of the median of the distribution from the mean value, and positive skewness indicates that the right tail of the distribution is flatter or longer than the left tail [12]. Kurtosis reflects the peakness of the histogram distribution, and high kurtosis tends to have a distinctive peak near the mean, and to decline rather rapidly and have heavy tails [12].

### Statistical analyses

All statistical analyses were done with PASW statistical software (ver. 23.0, SPSS, IBM, Chicago, IL). A *p*-value <0.05 was considered to indicate a significant difference. All measurements were correlated, and the concordance of the interobserver variability was tested by calculating Spearman and interclass correlation coefficients (ICCs). Agreement was interpreted according to the ICC as: > 0.8, excellent; 0.6–0.8, good; 0.4–0.6, moderate; and <0.4, poor concordant. We used the Jonkheere-Terpstra test to correlate the histogram parameters with the pathological grades of the tumors (grades 1–3). The Mann-Whitney test was used to compare the histogram parameters between high-grade (grade 3) and low-grade (grades 1 and 2) tumors. We also used the Mann-Whitney test to examine the correlations between the presence or absence of lymphovascular invasion and pleural invasion with each histogram parameter. We performed a receiver-operating characteristics (ROC) curve analysis to assess the diagnostic performance of histogram parameters for distinguishing different pathological features when appropriate.

## Results

### Reproducibility of the ADC measurements

Based on the ICC, the interobserver variability in the ADC measurements showed good to excellent agreement (0.78–0.99).

### Differentiation of the pathological grade of NSCLC using ADC histogram parameters

The mean and the 10th, 25th, 50th, 75th, 90th, and 95th percentiles of ADC values showed significant differences among the three pathological NSCLC grades (p = 0.000, 0.013, 0.001, 0.000, 0.000, 0.000, and 0.000, respectively) (Table 2).

**Table 2:** ADC Histogram Parameters of Pathologic Grades of NSCLC.

Parameter	Grade 1	Grade 2	Grade 3	p-value
	(n = 15)	(n = 30)	(n = 15)	
Mean	1411.4 (194.1)	1244.0 (154.3)	1121.0 (237.0)	0.000*
5th percentile	900.7 (192.8)	830.5 (173.4)	778.4 (199.6)	0.067
10th percentile	994.7 (182.8)	912.3 (150.8)	849.1 (202.6)	0.013*
25th percentile	1183.8 (186.7)	1035.9 (146.1)	960.2 (203.6)	0.001*
50th percentile	1379.1 (213.4)	1188.1 (148.4)	1090.8 (223.6)	0.000*
75th percentile	1606.1 (217.4)	1404.0 (177.4)	1251.2 (286.7)	0.000*
90th percentile	1881.3 (239.9)	1656.5 (260.2)	1445.3 (356.5)	0.000*
95th percentile	2041.1 (268.3)	1855.4 (314.5)	1571.0 (385.3)	0.000*
Skewness	0.56 (0.82)	0.93 (0.67)	0.76 (0.68)	0.309
Kurtosis	1.48 (2.65)	2.48 (2.93)	2.32 (2.34)	0.16

Values presented as mean (±SD)×10^−6^ mm^2^/sec).

*p<0.05 (Jonkheere-Terpstra test).

Other histogram parameters, including the 5th percentile of ADC, skewness, and kurtosis were not significantly different among the pathological grades.

### Differentiation of high- and low-grade NSCLC tumors using ADC histogram parameters

The mean and the 50th, 75th, 90th, and 95th percentiles of ADC proved to be significant histogram parameters for differentiating high-grade (n = 15) from low-grade (n = 45) NSCLC tumors (p = 0.004, 0.029, 0.006, 0.002, 0.002, and 0.002, respectively) (Table 3).

**Table 3:** ADC Histogram Parameters of High- and Low-Grade of NSCLC.

Parameter	Low Grade	High Grade	p-value
	(n = 45)	(n = 15)	
Mean	1299.8 (184.5)	1121.0(211.7)	0.004*
5th percentile	853.9 (181.0)	778.4 (199.6)	0.235
10th percentile	939.7 (164.8)	849.1 (202.6)	0.114
25th percentile	1085.2 (173.6)	960.2 (203.6)	0.029*
50th percentile	1251.8 (194.4)	1090.8 (223.6)	0.006*
75th percentile	1471.4 (212.3)	1251.2 (286.7)	0.002*
90th percentile	1731.6 (272.9)	1445.3 (356.5)	0.002*
95th percentile	1917.3 (309.7)	1571.0 (385.3)	0.002*
Skewness	0.81 (0.74)	0.81 (0.74)	0.791
Kurtosis	2.15 (2.85)	2.32 (2.71)	0.463

Values presented as mean (±SD)×10^−6^ mm^2^/sec).

*p<0.05 (Mann-Whitney test).

Other histogram parameters, including the 5th, 10th, and 25th percentiles of ADC, skewness, and kurtosis were not significantly different between the high-and low-grade tumors.

### ROC analysis of ADC histogram parameters for predicting high-grade NSCLC

**Fig 2:**
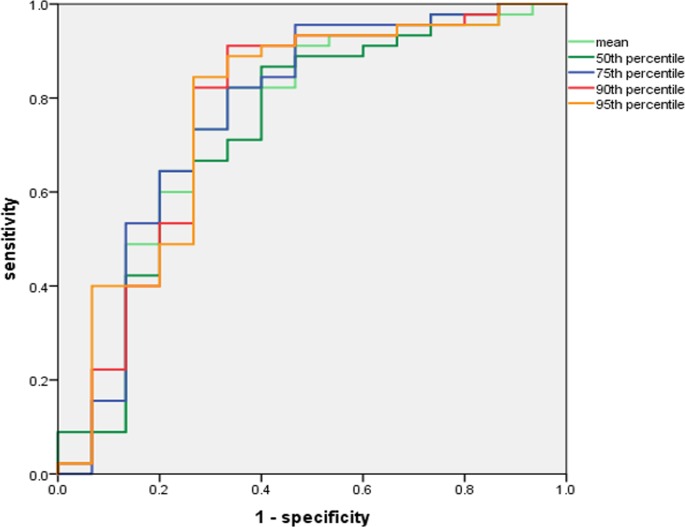
ROC curves of percentiles of ADC in predicting high-grade. The AUC was highest for the 95th percentile ADC (AUC = 0.74, cut-off value of 1634.1 × 10^−6^ mm^2^/sec, sensitivity 84.6%, specificity 66.7%).

The results of our ROC analysis of the histogram parameters between high- and low-grade tumors showed that the area under the curve (AUC) of the mean, 50th, 75th, 90th, and 95th percentiles of ADC were 0.706, 0.688, 0.713, 0.730, and 0.740, respectively. The 95th percentile ADC achieved the highest AUC, with a cut-off value of 1634.1 × 10^−6^ mm^2^/sec, 84.6% sensitivity, and 66.7% specificity (Fig 2).

### Differentiation of the presence or absence of lymphovascular invasion and pleural invasion using ADC histogram parameters

The presence of lymphovascular invasion was associated with the mean, 50th, 75th, 90th, and 95th percentiles of ADC, skewness, and kurtosis (p = 0.046, 0.033, 0.002, 0.003, 0.007, 0.02, and 0.001, respectively) (Table 4). Other histogram parameters, including the 5th, 10th, and 25th percentiles of ADC were not significantly different between the high- and low-grade groups. The presence of pleural invasion was associated only with skewness (p = 0.048) (Table 4). All of the other histogram parameters were not significantly different between the two groups.

**Table 4:** ADC Histogram Parameters of Lymphovascular Invasion and Pleural Invasion of NSCLC.

**Lymphovascular Invasion**			
**Parameter**	**Positive**	**Negative**	**p-value**
	(n = 49)	(n = 11)	
Mean	1227.1 (209.3)	1379.9 (182.2)	0.046*
5th percentile	839.4 (183.5)	815.6 (210.3)	0.474
10th percentile	916.0 (176.8)	921.8 (190.2)	0.841
25th percentile	1037.4 (180.0)	1127.6 (213.2)	0.293
50th percentile	1180.6 (197.0)	1349.5 (231.1)	0.033*
75th percentile	1371.3 (245.6)	1617.3 (155.1)	0.002*
90th percentile	1611.4 (325.9)	1876.6 (158.0)	0.003*
95th percentile	1780.2 (373.0)	2055.5 (170.5)	0.007*
Skewness	0.91 (0.69)	0.31 (0.67)	0.020*
Kurtosis	2.61 (2.83)	0.34 (0.69)	0.001*
**Pleural Invasion**			
**Parameter**	**Positive**	**Negative**	**p-value**
	(n = 30)	(n = 30)	
Mean	1253.4 (201.3)	1256.7 (225.1)	0.584
5th percentile	878.8 (155.7)	791.2 (207.3)	0.062
10th percentile	953.1 (152.7)	881.0 (195.4)	0.169
25th percentile	1064.1 (170.7)	1043.8 (206.0)	0.773
50th percentile	1202.7 (197.0)	1220.4 (229.2)	0.636
75th percentile	1397.9 (243.4)	1434.8 (258.6)	0.329
90th percentile	1642.2 (325.8)	1677.8 (315.2)	0.28
95th percentile	1804.1 (359.9)	1857.3 (364.3)	0.28
Skewness	0.97 (0.67)	0.62 (0.74)	0.048*
Kurtosis	2.49 (2.62)	1.90 (2.82)	0.069

Values presented as mean (±SD)×10^−6^ mm^2^/sec).

*p<0.05 (Mann-Whitney test).

### ROC analysis of ADC histogram parameters in predicting lymphovascular and pleural invasion

**Fig 3:**
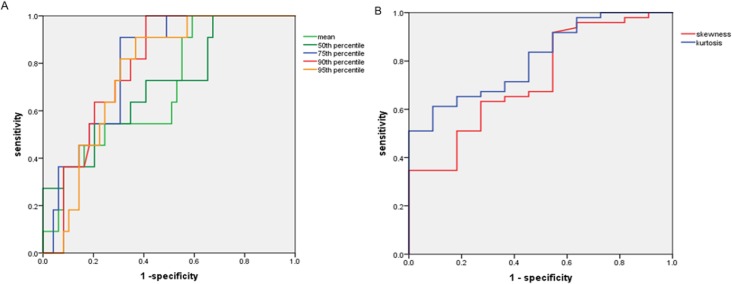
**ROC curves of percentiles of ADC (A), kurtosis and skewness (B) in predicting lymphovascular invasion.** The AUC was highest for the kurtosis (AUC = 0.809, cut-off value of 1.0815×10^-6^mm^2^/sec, sensitivity 61.2%, specificity 90.9%) in predicting lymphovascular invasion.

The ROC curve of the histogram parameters between the presence and absence of lymphovascular invasion showed that AUC values of the mean, 50th, 75th, 90th, and 95th percentiles of ADC, skewness, and kurtosis were 0.694, 0.707, 0.796, 0.788, 0.763, 0.726, and 0.809, respectively. Kurtosis achieved the highest AUC at 0.809, with a cut-off value of 1.0815 × 10^−6^ mm^2^/sec, with 61.2% sensitivity and 90.9% specificity (Fig 3).

**Fig 4:**
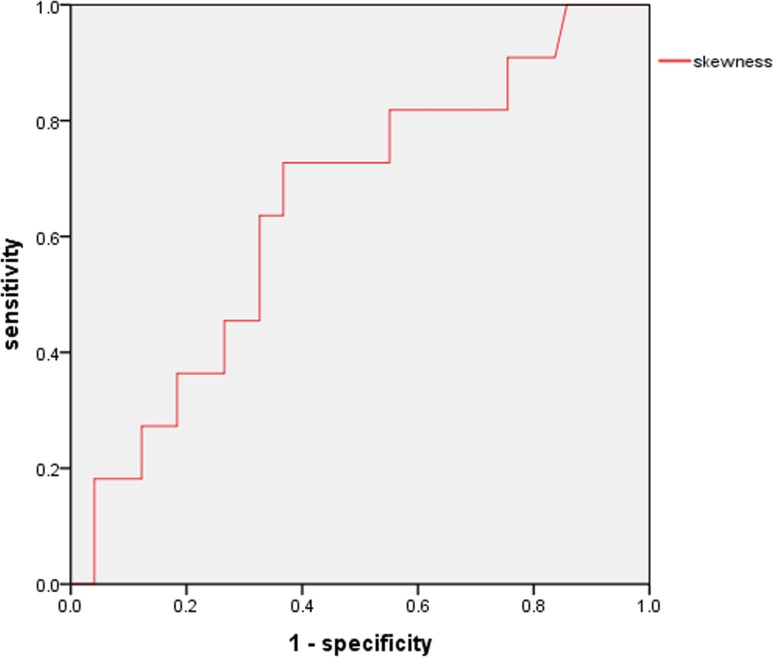
ROC curve of skewness in predicting pleural invasion. The AUC of the skewness was 0.648 (cut-off value 0.824×10^−6^ mm^2^/sec, sensitivity 60.0%, specificity 73.3%) in predicting pleural invasion.

For the skewness, according to the ROC curve, a cut-off value of 0.824 × 10^−6^ mm^2^/sec was associated with pleural invasion (AUC 0.648), with 60.0% sensitivity and 73.3% specificity (Fig 4).

## Discussion

DWI combined with ADC mapping has been investigated for use in lung cancer cases including mass-lesion detection, characterization, and to assess the patient's treatment response [6–11,15]. However, there are two problems to be addressed when considering DWI. First, the ADC values reported in these previous studies were obtained from a large ROI placed only on the largest lesion slice, and this may not reflect the characteristics of the entire tumor. Second, the mean ADC value was the only parameter that most studies adopted for lung cancer analysis. In the present study, we used the VOI encompassing the entire tumor to take into account the lesion texture and heterogeneity, using a histogram analysis.

To the best of our knowledge, no study has assessed the utility of ADC histograms for predicting the aggressiveness of lung cancer. The DWI of the lung has some problems, including (1) a low signal-to-noise ratio of the inherently low lung proton density, (2) distortion of the image due to cardiac and respiratory motion, and (3) magnetic susceptibility effects of the air-filled lung tissue subjected to large magnetic field gradients [4,5]. Two approaches have been used for DWI of the lung: breath-hold scanning and free-breathing scanning. Breath-hold scanning requires only a short examination time, but the signal-to-noise ratio is compromised especially at higher b-values, and this approach provides limited spatial resolution. Image acquisition during free breathing may be combined with cardiac triggering and/or respiratory triggering. Cardiac triggering is useful for avoiding pulsation artifacts, but is time-consuming [4]. The use of respiratory triggering improves the quality of DW images compared to the quality obtained using breath-hold imaging [16].

Various techniques have been used for monitoring the respiration such as a strain gauge, elastic breathing belts, and temperature monitoring using face masks, as well as navigator echoes [13,14,16]. The major advantage of the navigator-echo method is that no additional hardware is needed and the patient set-up is easier [13]. Taouli et al. reported that the use of a navigator-echo to trigger a single-shot EPI DWI sequence for the liver improves image quality and liver lesion conspicuity with more precise ADC measurement [14].

Similar to other studies of various types of malignant tumors [17–21], our present findings showed that all histogram-derived percentiles of the ADC based on the entire volume decreased as the tumor grade increased. All of the ADC percentile values except the 5th percentile significantly differentiated high- from low-grade tumors. Our ROC analysis revealed that the 95th ADC percentile is the most beneficial parameter for distinguishing high- from low-grade tumors (AUC 0.740). In addition, the AUCs of the mean, 75th, and 90th percentiles of ADC were >0.700.

The results of previous studies of the value of the percentile ADC in the differentiation of high- and low-grade malignancies have been controversial. In the studies of uterine cervical cancer, endometrial cancer, prostate cancer, bladder cancer, and brain glioma, low-percentile ADCs proved to be significant for differentiating high-grade from low-grade malignancies [18–20,22,23]. They stated that the low-percentile ADCs are correlated with highly cellular components in the tumor and is expected to decrease with higher grade, because high-grade tumors have higher cellularity and subsequently deceased extracellular space and diffusivity of water molecules.

On the contrary, in our present investigation, the high-percentile ADCs showed better diagnostic performance compared to that using low-percentile ADCs. Although the reason for this discrepancy remains unclear, one possible explanation is that high-grade lung cancers have much mucinous fluid, microhemorrhage, tissue disorganization—all of which contribute to reduce motion of water—and lower ADC values compared to low-grade lung cancers. As a result, these parts could be represented to a greater extent by high-percentile ADCs than by low-percentile ADCs.

A histogram-based analysis yields additional diffusion parameters regarding the distribution of ADC values, such as skewness and kurtosis [12]. Our present findings showed that the mean and the 50th, 75th, 90th, and 95th percentiles of ADC as well as skewness and kurtosis can be used to discriminate the presence of lymphovascular invasion. Among these parameters, kurtosis proved to be the most beneficial parameter in the ADC histogram (AUC 0.809). On the other hand, skewness was the only parameter that could discriminate the presence of pleural invasion (AUC 0.648). These results suggest that higher kurtosis and positive skewness are promising predictors of lymphovascular invasion and pleural invasion, respectively.

Apart from the intrinsic limitations of the retrospective nature of our study, several other limitations should be mentioned. First, the study population was relatively small. Further investigations that include larger populations are warranted to strengthen the statistical power of the results. Second, our study population did not include patients with predominant GGO, because the signal intensity of GGO was sometimes weak and could not be detected even in low-b value DWI [24,25]. Third, our study was performed using a 1.5-T MR system with two b-values (b = 0, 800 s/mm^2^) to acquire the DWI. The possibility of differing results using a higher magnetic field and a different number and magnitude of b-values cannot be excluded.

In conclusion, ADC histogram analyses on the basis of the entire tumor volume using respiratory triggering by the navigator-echo method are able to stratify the NSCLC’ tumor grade, lymphovascular invasion and pleural invasion. The 95th percentile ADC was the most promising parameter for the differentiation of high- from low-grade NSCLCs. The kurtosis and skewness were the predictive parameters for the presence of lymphovascular invasion and pleural invasion, respectively.

## Supporting information

S1 Fig Flowchart of patient selection process. (TIF)
