# Supplementary figures and images for: Non-small cell lung cancer: Whole-lesion histogram analysis of the apparent diffusion coefficient for assessment of tumor grade, lymphovascular invasion and pleural invasion
Source: PLoS One. 2017 Feb 16;12(2):e0172433. doi: 10.1371/journal.pone.0172433 (PMC5313135; doi:10.1371/journal.pone.0172433)

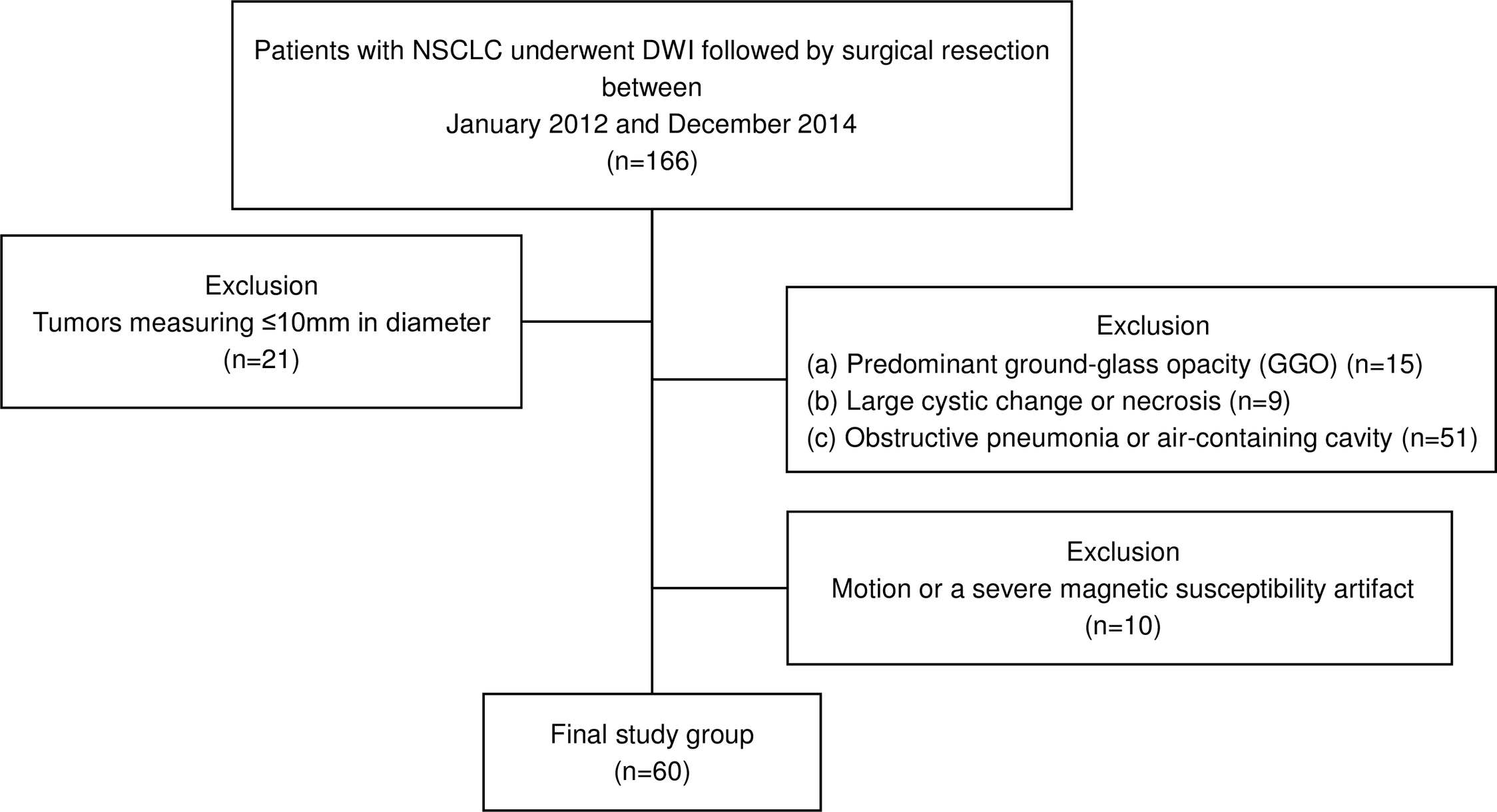

Supplement: S1 Fig — (TIF) [file pone.0172433.s001.tif]
